# Tannic Acid Mitigates Rotenone-Induced Dopaminergic Neurodegeneration by Inhibiting Inflammation, Oxidative Stress, Apoptosis, and Glutamate Toxicity in Rats

**DOI:** 10.3390/ijms24129876

**Published:** 2023-06-08

**Authors:** Sheikh Azimullah, Mohamed Fizur Nagoor Meeran, Khatija Ayoob, Seenipandi Arunachalam, Shreesh Ojha, Rami Beiram

**Affiliations:** Department of Pharmacology and Therapeutics, College of Medicine and Health Sciences, United Arab Emirates University, Al Ain P.O. Box 15551, United Arab Emirates; azim.sheikh@uaeu.ac.ae (S.A.); nagoormeeran1985@uaeu.ac.ae (M.F.N.M.); kparekh@uaeu.ac.ae (K.A.); seenipandi@uaeu.ac.ae (S.A.)

**Keywords:** Parkinson’s disease, inflammation, oxidative stress, autophagy, α-Glutamate, tannic acid, rotenone

## Abstract

Parkinson’s disease (PD), a movement disorder, is a neurodegenerative disease characterized by the degeneration of dopaminergic neurons in the substantia nigra pars compacta (SNpc) region of the brain. The etiopathogenesis of PD involves increased oxidative stress, augmented inflammation, impaired autophagy, accumulation of α-synuclein, and α-Glutamate neurotoxicity. The treatment of PD is limited and there is a lack of agents to prevent the disease/delay its progression and inhibit the onset of pathogenic events. Many agents of natural and synthetic origin have been investigated employing experimental models of PD, mimicking human PD. In the present study, we assessed the effect of tannic acid (TA) in a rodent model of PD induced by rotenone (ROT), a pesticide and an environmental toxin of natural origin reported to cause PD in agricultural workers and farmers. Rotenone (2.5 mg/kg/day, i.p.) was administered for 28 days, and TA (50 mg/kg, orally) was administered 30 min before ROT injections. The study results showed an increase in oxidative stress, as evidenced by the depletion of endogenous antioxidants and enhanced formation of lipid peroxidation products, along with the onset of inflammation following a rise in inflammatory mediators and proinflammatory cytokines. ROT injections have also augmented apoptosis, impaired autophagy, promoted synaptic loss, and perturbed α-Glutamate hyperpolarization in rats. ROT injections also induced the loss of dopaminergic neurons subsequent to the activation of microglia and astrocytes. However, TA treatment was observed to reduce lipid peroxidation, prevent loss of endogenous antioxidants, and inhibit the release and synthesis of proinflammatory cytokines, in addition to the favorable modulation of apoptosis and autophagic pathways. Treatment with TA also attenuated the activation of microglia and astrocytes along with preservation of dopaminergic neurons following reduced loss of dopaminergic neurodegeneration and inhibition of synaptic loss and α-Glutamate cytotoxicity. The effects of TA in ROT-induced PD were attributed to the antioxidant, anti-inflammatory, antiapoptotic, and neurogenesis properties. Based on the present study findings, it can be concluded that TA may be a promising novel therapeutic candidate for pharmaceutical as well as nutraceutical development owing to its neuroprotective properties in PD. Further regulatory toxicology and translational studies are suggested for future clinical usage in PD.

## 1. Introduction

Parkinson’s disease (PD) is a progressive neurodegenerative disease and it stands second after Alzheimer’s disease [1]. PD is characterized by numerous symptoms, such as bradykinesia, rigidity, postural abnormalities, uncontrolled movements, muscle rigidity, tremor, cognitive impairment, emotional and olfactory abnormalities due to functional impairment of motor neurons, and continual loss of dopaminergic neuronal cells in the substantial nigra pars compacta (SNPc) [2,3]. The key arbitrators in the pathogenesis of PD are inflammation [4,5], oxidative markers [6], lipid peroxidation, brain aging, mitochondrial dysfunction, and synaptic impairment [2,7,8].

The activation of microglia and astrocytes has been well-documented in PD following the release of the proinflammatory mediators interleukin-6 (IL-6), interleukin-IL-1 β (IL-1 β), tumor necrosis factor-alpha (TNF-α), and matrix metalloproteinase 9 (MMP9), as well as free oxygen radicals [4]. The persistent and prolonged activation of proinflammatory cytokines and excessive generation of free oxygen radicals by the microglia and astrocytes results in oxidative stress and neuroinflammation, the key pathogenic events that lead to the loss of dopaminergic neurons and the progression of symptoms of PD [9,10]. Microgliosis and astrogliosis mediate the synthesis of cyclooxygenase-2 (COX-2) and inducible nitric oxide synthase (iNOS), the precursor and regulator molecules, which play important roles in the causation of neuroinflammation and oxidative stress [11]. The enhanced formation of free oxygen radicals along with increased lipid peroxidation is evidenced by increased formation of lipid peroxidation product, malondialdehyde (MDA), and nitric oxide (NO) concentration, along with depletion of reduced glutathione (GSH), catalase (CAT), and superoxide dismutase (SOD).

The most unique pathologic characteristic of PD is the aggregation of α-synuclein, which is implicated in cell membrane function and membrane curvature, and thus affects synaptic trafficking and vesicle budding [12]. PD pathology is believed to be initiated by misfolding and aggregation of α-synuclein in the dopaminergic neuronal membrane, thus affecting cell trafficking and neuronal interactions [13,14]. Oxidative stress influences α-synuclein misfolding and eventually leads to membrane lipid peroxidation due to its helix structure nature and its presence in neuronal membranes. Neuroinflammatory mediators and reactive oxygen species (ROS) contribute to the accumulation of α-synuclein aggregation and reduced clearance, culminating in cellular toxicity. The misfolded α-synuclein protein and damaged synaptic trafficking results in enhanced accumulation of toxic molecules, that should be cleared following autophagy to protect the dopaminergic neurons [15].

Autophagy, the clearance auto-system in the absence of pathology, is pivotal in PD for the removal of α-synuclein misfolded protein, toxic aggregates in the synaptic junction, and degraded proteins to prevent the pathogenesis of PD. However, impairment in autophagy leads to the accumulation of α-synuclein, and other long-lived and degraded proteins that the autophagosome fails to remove. Autophagosome, cup-closed α-synuclein, and other misfolded proteins fuse with amphisome. The misfolded and other organelles are then cargo-fused with the lysosome to form an autophagic lysosome pathway (ALP) to degrade and recycle the proteins needed for neuronal function. The ALP impairment and neuronal homeostasis are the involved pathways, and they augment reciprocally [15].

In PD, the other pathway that is impaired is neuronal cellular homeostasis, and the main factor involved is the mammalian rapamycin (mTOR) pathway. Reports from PD patients demonstrate the loss or impairment of mTOR, which indicates the role of mTOR in the causation of dopaminergic neurodegeneration. The mTOR mechanism mitigates the phosphorylation of p70s6 kinase and eukaryotic initiation factor 4E binding protein 1 (4E-BP1). In experimental models of PD, such as 6-hydroxydopamine (6-OHDA), 1-methyl-4-phenylpyridinium ion (MPP^+^), and ROT, attenuation of mTOR signaling and downstream signaling of p70s6 kinase inactivation have been reported [16]. Neuroinflammatory factors, free radical species, α-synuclein accumulation, and diminished mTOR altogether result in apoptosis and augment degeneration of dopaminergic neurons.

The occurrence of apoptosis is balanced by the antiapoptotic protein, Bcl2 (B cell leukemia/lymphoma 2), and the proapoptotic protein, Bax (Bcl2-associated X protein), which regulate the intrinsic apoptosis pathway by controlling the outer membrane permeability of the mitochondria. Under normal conditions, there is a delicate balance between apoptosis and autophagy to maintain neuronal homeostasis, and perturbations in this balance lead to the onset and progression of PD. Thus, in addition to targeting oxidative stress and inflammation, modulating apoptosis and autophagy represent novel therapeutic approaches for PD.

In the present study, we used rotenone (ROT), a pesticide and environmental neurotoxin of the rotenoid family, to induce PD in laboratory rats. Rotenone upon chronic exposure to agricultural workers, has been reported to cause PD and has been modeled to induce PD in rodents following different routes of administration. The rotenone-induced rodent model of PD is considered one of the appropriate models to represent the pathogenic features of PD, which are the degeneration of substantia nigra neurons and cytoplasmic inclusions, resembling Lewy bodies [17].

Currently, the drugs used in the treatment of PD solely rely on the agonism of dopamine, the inhibition of cholinergic receptors, and the inhibition of monoamine oxidase and catechol-o-methyltransferase enzymes, which inactivate dopamine [18]. The diagnosis of PD is often made when more than 60–80% of dopaminergic neurons are already lost. The available drugs only provide symptomatic relief, with numerous adverse drug reactions. Therefore, newer, and better drugs are needed, which may delay, check, or halt the progression of symptoms. As discussed earlier, PD involves multiple mechanisms, including oxidative stress, inflammation, apoptosis, and autophagy, the agents which could target all these pathogenic targets may have value in preventive therapy.

In recent years, numerous phytochemicals, plant-derived secondary metabolites possessing pleiotropic effects, and considered relatively safe over synthetics with negligible toxicity, have received attention for their therapeutic and preventive properties in PD. In the numerous phytochemical classes, polyphenolic compounds are of enormous importance due to their dietary availability and multiple health benefits with pharmacological effects. One of the polyphenolic compounds, tannic acid (TA), chemically, hydrolyzable tannin polyphenols, has received wide recognition due to its abundant presence in many edible plants, including grapes, tea, gall nuts, hazelnuts, and walnuts, with major concentrations in seeds, bark, cones, and heartwood [19,20]. Tannic acid has been reported to mitigate oxidative stress and inflammation, and modulate apoptosis and autophagy, in numerous studies by modulating cell signaling pathways [21]. Additionally, TA has also been shown to possess anticarcinogenic, antimutagenic, antitumor, and antimicrobial activities. The LD_50_ of TA in rats following the oral route is 5 g/kg [22]. A convincing number of studies have demonstrated that plants rich in TA were shown beneficial in neurodegenerative diseases [21]. However, there is no systematic scientific data on the role of TA in PD. Recently, TA has been shown to reduce Tau aggregation in AD [23].

In the present study, we evaluated the role of TA in the ROT-induced rat model of PD and elucidated the underlying mechanisms. The parameters of oxidative stress, inflammation, apoptosis, and autophagy were determined, in addition to the assessment of synapse impairment and dopaminergic neurodegeneration.

## 2. Results

### 2.1. Tannic Acid Mitigated the ROS Induced by Rotenone

**Figure 1:**
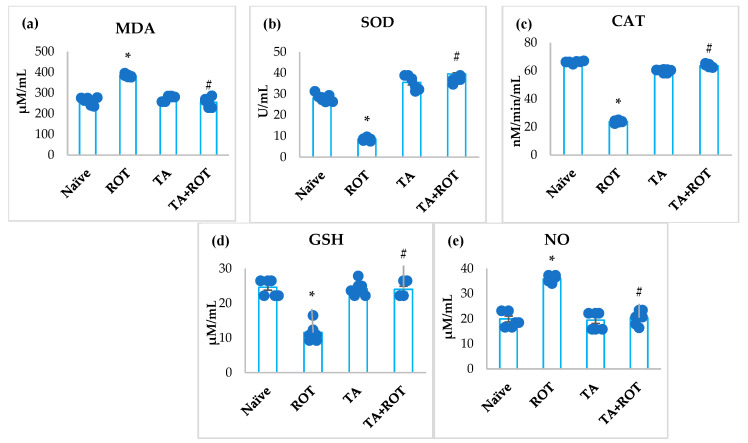
Effect of tannic acid on the levels of MDA, SOD, CAT, GSH, and NO in the midbrain of rats (**a**–**e**). The values are presented as mean ± SEM (*n* = 7). * *p* < 0.05 CON vs. ROT; # *p* < 0.05 ROT vs. TA + ROT (one-way ANOVA followed by DMRT). CON: normal control, ROT: rotenone, TA: tannic acid, TA + ROT: tannic acid and rotenone.

Rotenone injections caused depletion of antioxidants, SOD, CAT, and GSH, along with an abrupt increase in the levels of MDA and NO (Figure 1a–e). Whereas treatment with TA produced a significant (*p* < 0.05) decrease in MDA and NO, and prevented the depletion of GSH, SOD, and CAT in the ROT-administered group (Figure 1a–e).

### 2.2. Tannic Acid Diminished Upregulation of Proinflammatory Markers and Metallopeptidases (MMP9)

**Figure 2:**
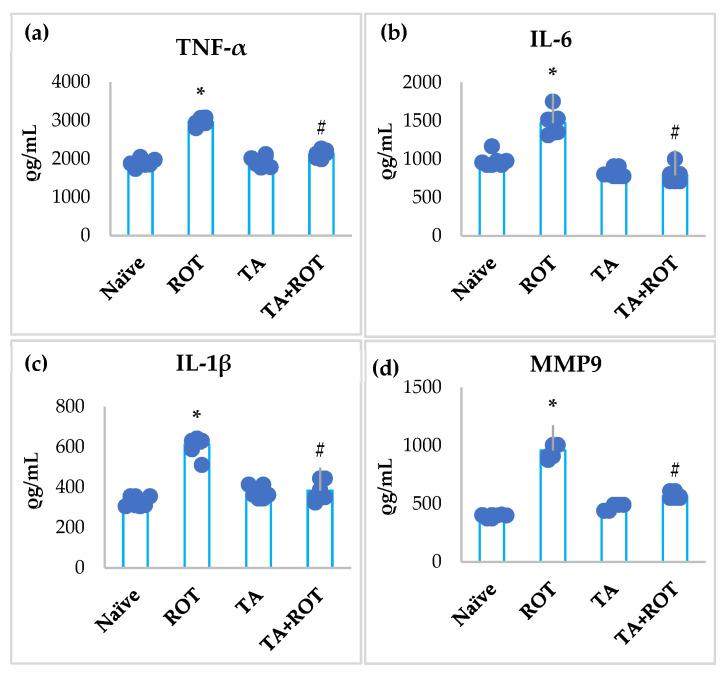
Effect of tannic acid on the levels of TNF-α, IL-6, IL1β, and MMP9 in the midbrain of rats (**a**–**d**). The values are presented as mean ± SEM (*n* = 7). * *p* < 0.05; CON vs. ROT; # *p* < 0.05 ROT vs. TA + ROT (one-way ANOVA followed by DMRT). CON: normal control, ROT: rotenone, TA: tannic acid, TA + ROT: tannic acid and rotenone.

**Figure 3:**
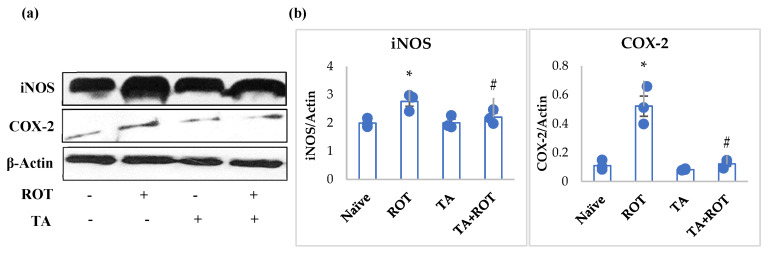
Immunoblotting analysis and quantification of iNOS and COX-2 in the striatal tissues of rats (**a**,**b**). Immunoblotting was performed in triplicates. The results are presented as mean ± SEM (*n* = 3). * *p* < 0.05 CON vs. ROT; # *p* < 0.05 ROT vs. TA + ROT (one-way ANOVA followed by DMRT). CON: normal control, ROT: rotenone, TA: tannic acid, TA + ROT: tannic acid and rotenone.

A significant increase in proinflammatory cytokines (IL-6, IL-1 β, TNF-α) and MMP9 was observed in ROT-injected rats (Figure 2a–d). However, treatment with TA significantly reduced the increase in the levels of IL-6, IL-1 β, TNF-α, and MMP9 (Figure 2a–d). Rats administered ROT showed a significant increase in the expression of COX-2 and iNOS, regulators of inflammation and oxidative stress, respectively, whereas treatment with TA mitigated overexpression of COX-2 and iNOS (Figure 3a,b).

### 2.3. Tannic Acid Reinstated the Activation of Microglia and Astrocytes

**Figure 4:**
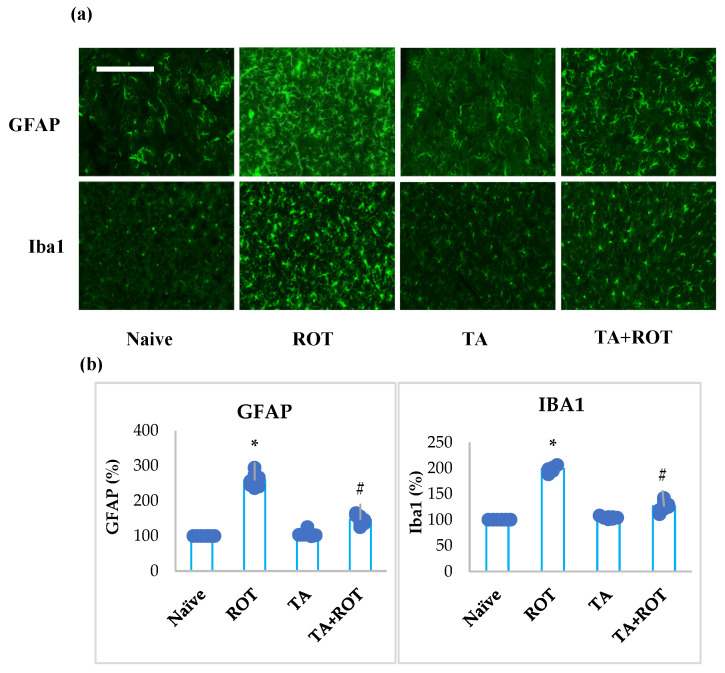
The activation of GFAP and Iba-1 in the striatum was determined by immunofluorescence staining of the striatum. A remarkable expression of activated astrocytes (GFAP-positive) and microglia (Iba-1-positive) (**a**) was observed in the fluorescent images taken from ROT-injected rats when compared to the control rats. (**b**) The quantitative analysis of the number of activated astrocytes and microglia is shown. Each group consists of three rats and the values are presented as mean ± SEM (n = 7 images). * *p* < 0.05 CON vs. ROT; # *p* < 0.05 ROT vs. TA + ROT (one-way ANOVA followed by DMRT). CON: normal control, ROT: rotenone, TA: tannic acid, TA + ROT: tannic acid + rotenone. Magnification: 20×.

The persistent activation of microglia and astrocytes is a key factor in neuroinflammation subsequent to oxidative stress. The microglia and astrocytes were stained with Iba-1 and GFAP, respectively (Figure 4a,b). Rats injected ROT showed significant activation of microglia and astrocytes when compared to the normal control group. However, TA treatment in the ROT-injected rats showed a significant reduction in the activation of microglia and astrocytes, as evidenced by IBa-1 and GFAP staining (Figure 4a,b).

### 2.4. Tannic Acid Prevented the Loss of Dopaminergic Neurons

**Figure 5:**
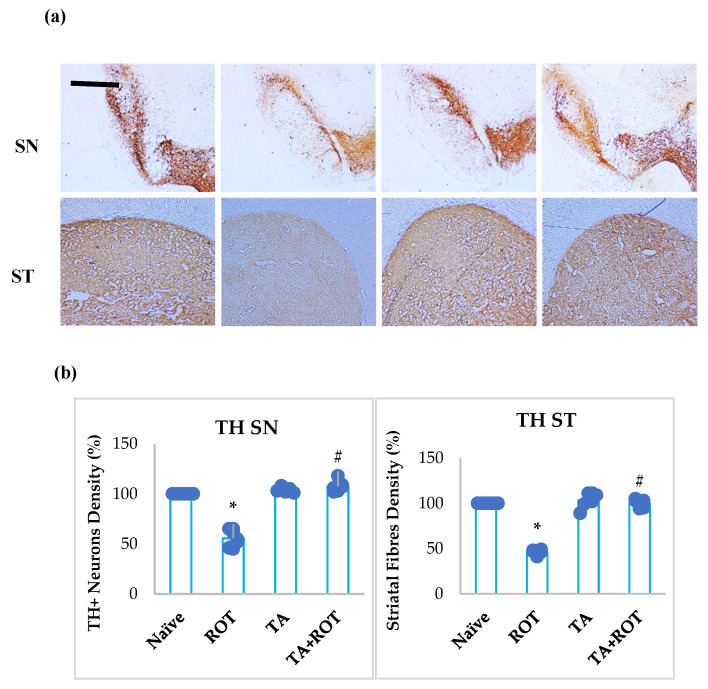
TH-ir neurons and TH-ir fibers in the substantia nigra (SN) and striatum are presented, respectively (**a**) (scale bar is 20 µm). (**b**) Quantification of the TH-ir neurons in SNc and the density of TH-ir fibers are also shown. Each group contains three rats, and the data are represented as percent mean ± SEM (*n* = 7 images). * *p* < 0.05 CON vs. ROT; # *p* < 0.05 ROT vs. TA + ROT (one-way ANOVA followed by DMRT). CON: normal control, ROT: rotenone, TA: tannic acid, TA + ROT: tannic acid + rotenone.

The enzyme tyrosine hydroxylase is a rate-limiting enzyme involved in the synthesis of dopamine. The inhibition of tyrosine hydroxylase-positive neurons was quantified in the striatum, as evidenced by a significant reduction in TH expression (Figure 5a,b). However, TA treatment to ROT-injected animals significantly prevented the loss of dopaminergic neurons in SNPc and striatal TH-positive dopaminergic neuronal projections compared to the normal control group (Figure 5a,b).

### 2.5. Tannic Acid Attenuated Overexpression of α-Synuclein, a Hallmark of PD

**Figure 6:**
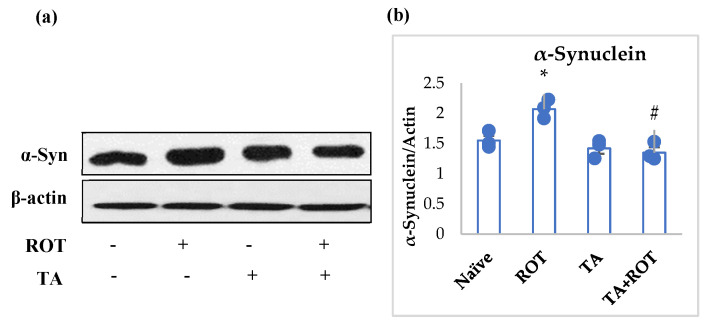
Western immunoblotting and densitometric analysis of the protein expression of α-synuclein in the striatal tissues of rats (**a**,**b**). Immunoblotting was performed in duplicates and the values are presented as mean ± SEM (*n* = 3). * *p* < 0.05 CON vs. ROT; # *p* < 0.05 ROT vs. TA + ROT (one-way ANOVA followed by DMRT). CON: normal control, ROT: rotenone, TA: tannic acid, TA + ROT: tannic acid + rotenone.

The aggregation of α-synuclein is the hallmark of PD pathogenesis. Rotenone injections significantly induced overexpression of α-synuclein, which could be indicative of α-synuclein accumulation. However, treatment with TA was observed to significantly reduce the expression of α-synuclein in the striatum of rats when compared to the ROT control group (Figure 6a,b).

### 2.6. Tannic Acid Reinstated Autophagic Flux and Autophagosome Vesicles

**Figure 7:**
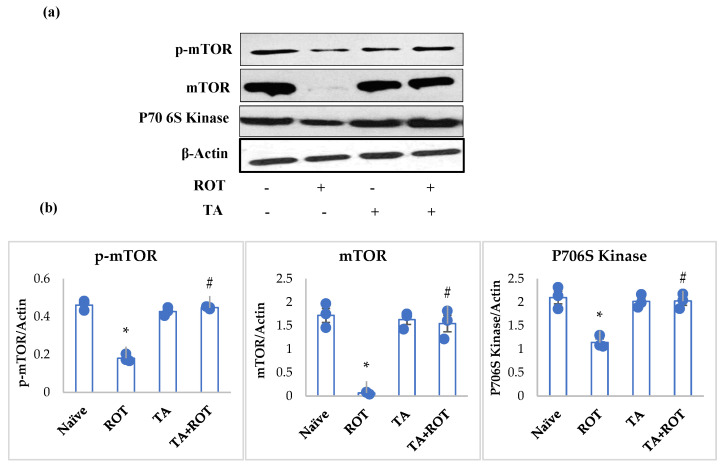
Western immunoblotting and densitometric analysis of the protein expression of p-mTOR, mTOR, and p70s6 kinase in the striatal tissues of rats (**a**,**b**). Immunoblotting was performed in duplicates and the values are presented as mean ± SEM (*n* = 3). * *p* < 0.05 CON vs. ROT; # *p* < 0.05 ROT vs. TA + ROT (one-way ANOVA followed by DMRT). CON: normal control, ROT: rotenone, TA: tannic acid, TA + ROT: tannic acid + rotenone.

**Figure 8:**
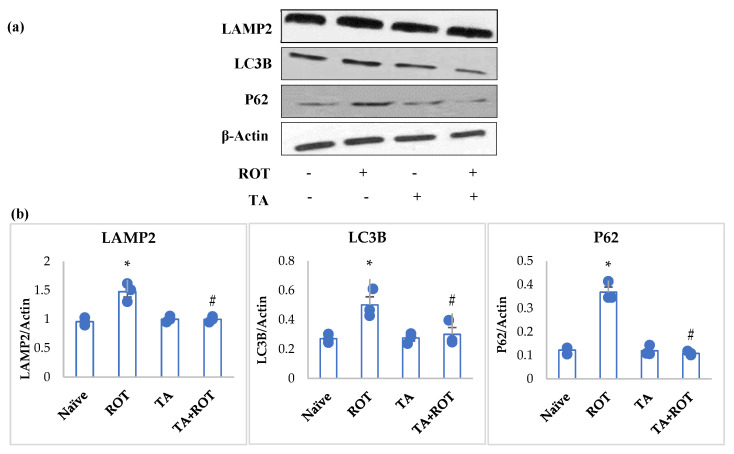
Western immunoblotting and densitometric analysis of the protein expression of LAMP2, LC3B, and P62 in the striatal tissues of rats (**a**,**b**). Immunoblotting was performed in duplicates and the values are presented as mean ± SEM (*n* = 3). * *p* < 0.05 CON vs. ROT; # *p* < 0.05 ROT vs. TA + ROT (one-way ANOVA followed by DMRT). CON: normal control, ROT: rotenone, TA: tannic acid, TA + ROT: tannic acid + rotenone.

PD has been shown to be associated with impaired autophagic flux, which is attributed to the impairment of autophagosome vesicles. Rats injected with ROT exhibited a significant decline in the expression of markers of autophagy: mTOR, p-mTOR, and p70s6 kinase (Figure 7), and activation in autophagosome vesicles: LAMP2, P62, and LC3II (Figure 8a,b). Whereas TA treatment to ROT-injected rats showed significant improvement in the mediators of autophagy (Figure 7) and downregulated the expression of LAMP2, P62, and LC3II, the markers of autophagosome vesicles (Figure 8a,b).

### 2.7. Tannic Acid Reinstated the Synapses by Inhibiting the NMDR Receptors

**Figure 9:**
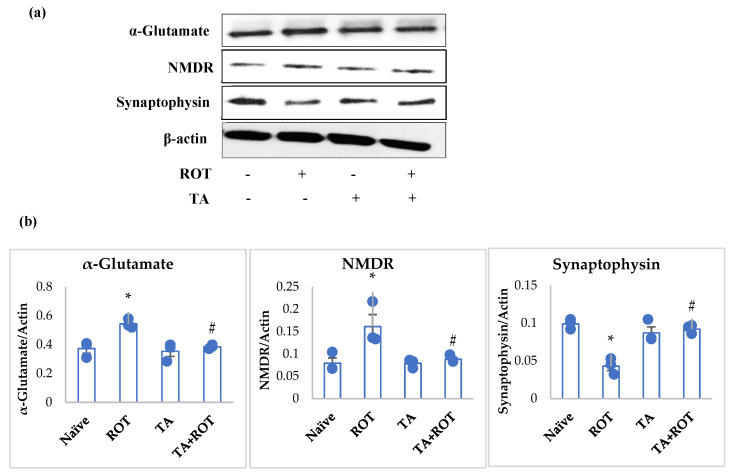
Western immunoblotting and densitometric analysis of the protein expression of α-Glutamate, NMDR, and synaptophysin in the striatal tissues of rats (**a**,**b**). Immunoblotting was performed in duplicates and the values are presented as mean ± SEM (*n* = 3). * *p* < 0.05 CON vs. ROT; # *p* < 0.05 ROT vs. TA + ROT (one-way ANOVA followed by DMRT). CON: normal control, ROT: rotenone, TA: tannic acid, TA + ROT: tannic acid + rotenone.

Rats injected with ROT showed a significant increase in the expression of α-Glutamate and NMDR, along with a significant decrease in synaptophysin (Figure 9a,b). However, rats received TA treatment showed significant downregulation of α-Glutamate, NMDR, and upregulation of synaptophysin (Figure 9a,b).

### 2.8. Tannic Acid Mitigated Apoptosis Induced by ROT

**Figure 10:**
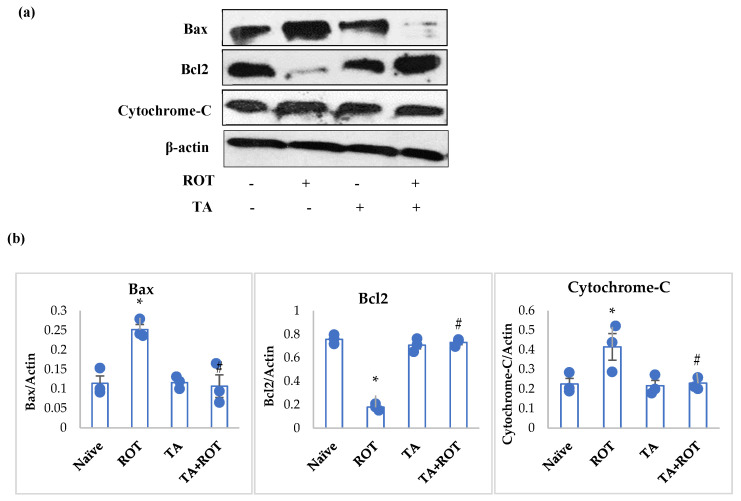
Western immunoblotting and densitometric analysis on the protein expression of Bax, Bcl2, and cytochrome-C in the striatal tissues of rats (**a**,**b**). Immunoblotting was performed in duplicates and the values are presented as mean ± SEM (*n* = 3). * *p* < 0.05 CON vs. ROT; # *p* < 0.05 ROT vs. TA + ROT (one-way ANOVA followed by DMRT). CON: normal control, ROT: rotenone, TA: tannic acid, TA + ROT: tannic acid + rotenone.

Rotenone-injected rats showed a significant increase in Bax and cytochrome-C, concomitant with a significant decrease in Bcl2 (Figure 10a,b). However, TA treatment significantly mitigated the overexpression of Bax and cytochrome-C and upregulated the expression of Bcl2 (Figure 10a,b).

## 3. Discussion

In the present study, we evaluated the neuroprotective role of TA in a rodent model of ROT-induced experimental model of PD exhibiting dopaminergic neurodegeneration, mimicking human PD. The neuroprotective effect of TA was evidenced by the inhibition of lipid peroxidation and oxidative stress, along with suppression of inflammatory mediators, followed by preservation of dopaminergic neurons and reduced activation of microglia and astrocytes. TA treatment also improved autophagy and reduced apoptosis, along with reducing the expression of synuclein, a pathologic hallmark of PD.

Rotenone is a natural herbicide used in agriculture and it is classified as a hazardous compound upon inhalation or ingestion. It is lipophilic, crosses the BBB, and leads to the onset and development of PD. The incidence of PD is 2.5 times higher in ROT-exposed than in nonexposed individuals [24]. The establishment of PD following ROT injections in rats is one of the popular models to evaluate drugs for their therapeutic and preventive potential in PD. Rotenone is a well-known inhibitor of mitochondrial complex 1 that results in mitochondrial impairment, increased formation of ROS, and induction of neuroinflammation, followed by the activation of astrocytes and microglia, along with dopaminergic neuronal loss, apoptosis, and impaired autophagy.

Being an inhibitor of mitochondrial complex-1, ROT alters cellular homeostasis by engendering energy to neuronal cells through oxidative phosphorylation and enhanced oxidative stress [25]. Oxidative stress involves an imbalance of ROS generation by the phosphorylation, the release of an unpaired electron-produced superoxide radical (O_2_), converted by mitochondrial superoxide dismutase to H_2_O_2_ in the mitochondria, outfits in the cytosol and nucleus, and ultimately culminates in neuronal injury [26]. Inhibition of mitochondrial complex-1 and the subsequent increase in ROS became the main arbitrator of dopaminergic neuronal damage. Additionally, activation of microglia and astrocytes, along with neuroinflammation, causes dopaminergic neuronal loss in PD. The neuronal cells are vulnerable to oxidative stress due to their high lipid content and energy requirements [27]. In the present study, MDA, a marker of lipid peroxidation, and CAT, SOD, GSH, and NO, were measured to determine the occurrence of oxidative markers. The results revealed that ROT injections induced enhanced formation of the lipid peroxidation product, and depletion of GSH, CAT, and SOD were attenuated by TA, as evidenced by reduced MDA, along with restoration of SOD, CAT, and GSH, which indicates the anti-lipid-peroxidative and antioxidant properties of TA in the brain. Interestingly, TA was reported to inhibit vital enzymes such as xanthine oxidase and p67 phox, an NADPH oxidase subunit involved in superoxide anion overproduction. This clearly revealed the beneficial role of TA in reducing free radical-mediated oxidative stress, and it has been ascribed as a primary mechanism behind its neuroprotective potential against ROT-induced PD [28].

Mounted ROS facilitates neuroinflammation in PD through disruption of the BBB, amplified microgliosis, and infiltration of peripheral immune cells. The activation of microglia results in the upregulation of proinflammatory cytokines and MMPs. Inflammation is an encompassing term for the multifaceted immune responses to neuronal cell damage, toxins, and abnormal protein clearance [29]. The central nervous system response to immune insults is orchestrated through microglia activation, astrocytes, and oligodendrocytes [3]. Microglia, in the healthy brain, perform a scavenging role, removing debris and excess factual molecules, following neurological insults when chronically activated. Activated microglia is a major source of ROS in the state of neuroinflammation. As mentioned earlier, dopaminergic neurons are susceptible to ROS, encompass reduced antioxidant enzymes, and thus initiate irregular neuronal cell signaling, degeneration, and death of neuronal cells [30]. Activated microglia, on the one hand, produce proinflammatory cytokines, causing neurotoxicity, and on the other hand, they release chemoattractant and MMPs, causing infiltration of leukocytes and damage to the BBB by MMPs, thus exacerbating inflammatory responses [31,32]. The self-perpetuating activation of microglia, and the subsequent rise of ROS, inflammatory cytokines, MMPs, and dopaminergic neurons, enduring chemoattractant and further activated microglia, increased neuroinflammation that resulted in augmented dopaminergic neuronal loss [3]. Following treatment with TA, the diminution of the activation of microglia and astrocytes demonstrated the restorative effects of TA on astrocytes and microglia.

In addition to oxidative stress, persistent, low-grade, chronic neuroinflammation is one of the main culprits in PD progression. Neuroinflammation is considered a “double-word-edge”, which on one hand clears the toxins and unwanted molecules, and on the other hand, sustained rise in the proinflammatory factors contribute to the PD pathogenicity [4]. The inhibition of mitochondrial complex-1, dysfunctional mitochondria, ROS production by microglia, oxidative alteration of α-synuclein and tyrosine hydroxylase, and upregulation of proinflammatory cytokines exert damaging effects on the neuronal cells, eventually resulting in dopaminergic neuronal cell loss [33,34]. The presence of inclusions of intraneuronal α-synuclein in PD brains is associated with PD and dementia with Lewy bodies [14,35]. The overexpression of α-synuclein, which is pivotal in enhancing neuroinflammation in PD, has been well-documented [14]. Studies have shown evidence of α-synuclein, mainly large oligomers/protofibrils in Lewy bodies. Thus, to normalize the neurotoxic properties, sustained neuroinflammation by the activated microglia and astrocytes has a profound detrimental effect on neuronal homeostasis and interactions [14,36]. In matrix metalloproteinases (MMPs), especially MMP9 is crucial to the BBB integrity. An increase in proinflammatory cytokines (IL-6, IL-1β, TNF-α) and MMP9 disrupts the maintenance of dopaminergic neurons and the function [37,38]. Previously, we showed that ROT induced proinflammatory cytokines in the striatum and substantia nigra [39]. The increase in proinflammatory cytokines and MMP9 in the ROT-induced group was attenuated by TA treatment. Further, TA was also found to reduce the expression of iNOS and COX-2 enzymes, which were upregulated following ROT administration. Collectively, the reduction in proinflammatory cytokines and enzymes demonstrated the anti-inflammatory effect of TA, which is in line with previous studies wherein TA inhibited the transcriptional activity of NF-κB and other downstream inflammatory signaling cascades related to the oxidative stress-induced inflammatory responses [21].

It is evident that neuroinflammation and oxidative stress trigger toxicity, accumulation of undesirable proteins, and α-synuclein aggregation [40,41]. The cumulative toxicity of these mediators is detrimental to PD and they need to be removed from neuronal cells; hence, the hindrance and failure to clear these culprits leads to the progression of PD and neuronal impaired growth, and eventually, neuronal death by initiating apoptosis [42]. The mTOR signaling pathway is the main pathway involved in homeostasis, cellular growth, and survival. mTOR is a serine/threonine protein that mediates phosphorylation of p70s6 kinase (p70s6K) and eukaryotic initiation factor 4E (eIF4E) binding protein 1 (4E-BP1). Impairment in mTOR results in dopaminergic suppression and lessens memory formation [43,44,45]. It is reported that inhibition of mTOR induces autophagy for survival and eventually increases the autophagic burden by limiting the autophagic outflux of vital proteins for recycling, also limiting the toxic factors and thus impairing cellular growth [46]. Similarly, the delay in the removal of aggravated and toxic molecules results in enhanced toxicity and dopaminergic loss, ascribed to the impaired autophagy, which is associated with the clampdown of dysfunctional lysosomal-autophagosome [47].

The present study results showed ROT-induced inhibition of the mTOR-mediated pathway in the PD model. The mTOR, p-mTOR, and p70s6 kinase showed low expression in the ROT group compared to the normal group, indicating neuronal impaired homeostasis. However, TA treatment appeared to alleviate neuronal homeostasis by reinstating the mTOR pathway. Additionally, we analyzed the autophagic factors, lysosomal-associated membrane protein type 2A (LAMP2) and microtubule-associated protein 1A/1B light chain (LC3), to evaluate the α-synuclein and other toxic molecules in the synaptic vesicles’ clearance by autophagy. The suppression of LAMP-2A, an arbitrator of α-synuclein-binding at the lysosomal membrane, altered the autophagic machinery and failed to eradicate the α-synuclein and other unwanted proteins [48,49]. Other factors, such as LC3 and p62, are responsible for the buildup of unsought proteins’ elimination, increased in PD, that impaired autophagosme formation, thus mitigating the autophagic function and inducing apoptosis [50,51,52]. Our results showed a marked decrease in the LAMP2, and increased expression of LC3b and p62 following rotenone administration, while TA treatment when administered to ROT-challenged rats increased LAMP2 expression and mitigated LC3b and p62 expressions.

In a normal physiological state, α-synuclein mediates the neuronal transmitter release and inhibits the upregulation of neurotransmitters localized in the nerve terminals [12,53]. The presence of α-synuclein in the synaptic vesicles and the mediation of neurotransmitters are altered when α-synuclein misfolding initiates, culminating in toxic oligomers and causing neuronal toxicity. The loss of dopaminergic neurons in the substantia nigra accompanied by α-synuclein in Lewy bodies in PD is associated with energy depletion due to mitochondrial complex-1 inhibition [2,54]. Synaptic loss in PD and frontotemporal dementia is associated with α-synuclein oligomerization. The mechanism implicated in synaptic loss is the aggregation and oligomerization of α-synuclein, which induce a plethora of α-Glutamate not only in neurons but also in microglia and astrocytes [8]. It was evidenced that the release of Ca^+2^ ions and α-Glutamate from astrocytes and microglia resulted in a plethora of α-Glutamate in synaptic vesicles, triggering NMDA receptor expressions and contributing to synaptic impairment, and hence PD progression [55]. The present study results for the synaptic vesicles markers reflected excitotoxicity, as evidenced by mounted α-Glutamate release, abundant expression of NMDA receptors, and diminished synaptophysin and neuronal impairment following ROT injections. However, TA treatment was found to restore the synaptic function by attenuating α-Glutamate release, lessening NMDA receptors expression, and reinstating synaptophysin.

α-Synuclein aggregation in synaptic vesicles induces apoptosis by the NMDA receptor upregulation and α-Glutamate release. α-Glutamate overexpression induced following α-synuclein expression is abrogated by dopamine and antioxidant molecules [10]. Other factors that induce apoptosis in dopaminergic neurons are neuroinflammation [56], oxidative stress [27,57], and autophagy [4]. These complement each other and alter the ratio of Bax/Bcl-2 in dopaminergic neurons, leading to the onset of apoptosis. The mechanism of intrinsic apoptosis in PD follows the release of cytochrome-C in cytosol, which is pivotal to α-synuclein’s radical formation and oligomerization, resulting in dopaminergic neuronal death [58]. The present study results showed increased apoptosis in dopaminergic neurons by upregulating Bax and diminishing Bcl2 expression after ROT administration. TA treatment reduced apoptosis, as evidenced by favorable modulation of Bcl-2, Bax, and cytochrome-C. Previously, it was demonstrated that ROS inhibition considerably suppress the expressions of apoptotic proteins [59], and thus, TA, as a potent antioxidant, invoked its neuroprotective actions through its potent free radical scavenging and antioxidant properties.

Based on the present study findings, it can be concluded that TA has the potential to exert a protective effect against ROT-induced dopaminergic neurodegeneration by mitigating oxidative stress and neuroinflammation, correcting the impaired energy-dependent mediators, along with restoring autophagy and attenuating α-Glutamate cytotoxicity, as well as apoptosis. Tannic acid was also observed to preserve the dopaminergic neurons and impede NMDA receptor upregulation, which provided resistance to neuronal communication. Further studies on pharmacokinetics and regulatory toxicology are suggested for the pharmaceutical as well as nutraceutical development of tannic acid.

### Strengths and Limitations of the Study

The present study findings clearly demonstrated the neuroprotective effects and underlying mechanism of TA in dopaminergic neurodegeneration. The study revealed that TA holds preventive and therapeutic promise against ROT-induced dopaminergic neurodegeneration in rats. The present findings could be encouraging to perform future studies to investigate the mechanistic aspect of TA on the assembly of α-synuclein. The effect of tannic acid can also be investigated in other animal models of PD including MPTP or 6-hydroxylamine-induced PD. Further, regulatory toxicology and pharmacokinetic studies are also suggested to encourage its development as a nutraceutical or phytopharmaceutical with a pharmacological rationale.

## 4. Materials and Methods

### 4.1. Experimental Animals and Ethical Approval

In the present study, Wistar male albino rats weighing 280–300 g (10–12 weeks old) were used. The original stock of Wistar rats was purchased from Harlan Laboratories (Harlan Laboratories, Oxon, England, UK). The animals were inbred in our animal facility from the original stock. The experimental rats used in the study were kept at 4 rats per cage, with a size of 43 × 22.5 × 20.5 cm^3^, in polypropylene cages. The food and water were available ad libitum. Prior to conducting the animal experiments, ethical approval (ERA_2021_7274, approved on 26 March 2021) was obtained from the Institutional Animal Ethics Committee of United Arab Emirates University. The rats were housed in the animal research facility under standard conditions of temperature (22 ± 1 °C), humidity (50–55%), and photoperiod (12/12 h). The animal experiments were carried out following approval from the Animal Ethics Committee of the United Arab Emirates University in accordance with the recommended criteria.

### 4.2. Chemicals and Reagents

Rotenone and TA of the highest purity were bought from Sigma Aldrich, Missouri, USA. The RIPA buffer and antibodies for glial fibrillary acidic protein (GFAP), COX-2, and iNOS were purchased from Sigma Aldrich, (St. Louis, MO, USA). The protease and phosphatase inhibitor combination were obtained from Thermo Fisher Scientific (Waltham, MA, USA). A polyclonal rabbit anti-tyrosine hydrolase antibody was obtained from Merck, Darmstadt, Germany. The antibodies, LC3, p62, mTOR, phosphor mTOR, and p70s6, were purchased from Cell Signaling Technology, (Danvers, MA, USA). The apoptotic polyclonal markers, Bax and Bcl-2, were purchased from (Cambridge, Abcam, MA, USA), and the monoclonal mouse anti-synuclein antibody was obtained from BD Biosciences, (San Jose, CA, USA). Anti-Iba-1 antibody was obtained from Wako Chemicals, Richmond, VA, USA. The fluorescent secondary antibody, Alexa Flour 488, was purchased from Thermo Fischer Scientific, (Waltham, MA, USA). The biotinylated secondary goat anti-rabbit antibody was obtained from Jackson immune research laboratory, (Baltimore Pike, West Grove, PA, USA). The biochemical assays were performed using commercially available kits. The additional compounds utilized in these tests were all purchased from regional vendors and were of analytical-grade quality.

### 4.3. Study Design and Experimental Protocol

For the induction of PD in rats, ROT injections (2.5 mg/kg, i.p.) were administered as standardized in our laboratory and described previously [6]. Tannic acid was administered orally at a dose of 50 mg/kg of body weight dissolved in water, while maintaining a pH of 7.0 to 7.5. Tannic acid was administered orally 30 min prior to the ROT injection for 28 days. The dose of TA (50 mg/kg) was selected based on a previous study [60]. The experimental animals were randomly assigned into four experimental groups: Group 1, assigned as the naive control group; Group 2, assigned as the ROT control group; Group 3, assigned as the TA control group; Group 4, assigned as the ROT + TA group. A total of 60 animals were used, divided into 15 animals/group. Eight animals were used for biochemical estimations (midbrain), four animals for Western blotting (striatum), and three animals were taken for immunohistochemistry (whole brain) per group. The body weight of the rats was recorded every week, including pre-dose and before termination of the experiment.

### 4.4. Tissue Handling Sample Preparation

Pentobarbital (40 mg/kg body weight) was administered intraperitoneally to anesthetize the rats, followed by intracardiac perfusion with phosphate-buffered saline (PBS 0.01 M). The brains were aseptically extracted for further assays and Western blotting, and others were fixed with 4% paraformaldehyde for the immunofluorescence assay. The striatal tissues were homogenized with RIPA buffer to perform Western blotting and the midbrain tissues were homogenized with KCL buffer (10 mM Tris-HCl, 140 mM NaCl, 300 mM KCl, 1 mM ethylenediaminetetraacetic acid, and 0.5% Triton-X100 at pH 7.5) to perform the enzyme-linked immunosorbent assay (ELISA) and other biochemical estimations.

### 4.5. Assessment of Malondialdehyde (MDA) Assay

The MDA detection kit was obtained from North-West Life Science (Vancouver, WA, USA) and the assay was performed according to the protocol provided with the kit. Briefly, 100 μL of the sample, 100 μL of calibrators, 100 μL of thiobarbituric acid, and 100 μL of acid reagents were dispensed in an Eppendorf tube, followed by 5 μL of BHT, and incubated for 1 h at 60 °C in an incubator for an optimal reaction. The reactants were centrifuged for 3 min at 850× *g* and the collected supernatant was transferred to a 96-well ELISA plate. The plate was read at 532 nm and the results were analyzed.

### 4.6. Estimation of Glutathione (GSH)

For the quantification of GSH, a commercially available GSH assay kit, acquired from Sigma-Aldrich Chemie GmbH, Steinheim, Germany, was used in the present study. The samples were deproteinized with 5% 5-sulfosalicylic acid. The sample (10 μL) or standard and 150 μL of the working mixture were added to the assay buffer, 5,5′-dithiobis (2-nitrobenzoic acid), and GSH reductase. Then, 50 µL of NADPH in a 96-well plate was added to the reaction mixture for optimal color development, and five kinetic readings were recorded in 6 min at 412 nm. The GSH content was analyzed and expressed as μM of GSH.

### 4.7. Determination of Antioxidant Enzymes: Superoxide Dismutase (SOD) and Catalase (CAT)

For the measurement of SOD, 10 μL of samples or the standard were dispensed in a 96-well plate, followed by 20 μL of xanthine oxidase to initiate the reaction. The plate was incubated at room temperature for 30 min. The SOD activity was then measured in U/mL following reading at 450 nm.

The activity of CAT was determined following the kit’s instructions, acquired from Cayman Chemicals Company (Ann Arbor, MI, USA). Briefly, in a 96-well plate, 20 μL of samples or the standard was added in designated wells, followed by methanol (30 μL) and assay buffer (100 μL). The reaction was initiated by adding 20 μL of hydrogen peroxide for 20 min, and optimal color development was achieved by dispensing 30 μL of potassium hydroxide to stop the reaction, 30 μL of CAT purpled, and terminating the reaction by adding 10 μL of potassium periodate. The results were quantified by reading the ELISA plate at 540 nm.

### 4.8. Estimation of Proinflammatory Cytokines and MMP9

The midbrain tissues were homogenized in KCL buffer (10 mM Tris-HCl, 140 mM NaCl, 300 mM KCl, 1 mM ethylenediaminetetraacetic acid, and 0.5% Triton-X100 at pH 7.5). The supernatants were used for ELISA and biochemical estimations. The midbrain was used because of the technical difficulty in the extraction of the substantia nigra. The midbrain represents the substantia nigra at large. The concentrations of proinflammatory cytokines (TNF-α, IL-1β, and IL-6) and MMP9 were quantified in midbrain tissues using commercially available kits (R&D Systems, Minneapolis, MN, USA). The plates were coated with respective antibodies by dispensing 100 μL and incubated for 24 h at room temperature. The plates were washed 3 times and 1% BSA was added to block the reaction. The secondary solution was dispensed in 100 μL and incubated for 2 h at room temperature. The plates were washed, and horse-reddish peroxide (HRP) was added to the plates in 100 μL after washing 3 times. The colorimetric optimization was achieved by adding 100 μL of TMB after washing 3 times, and the reaction was terminated by dispensing 50 μL of the stopping solution of 2N H_2_SO_4_. The results were analyzed and quantified at 450 nm absorbance using an ELISA reader. Absorbance was recorded at 450 nm, and the concentrations of pro-inflammatory cytokines and MMP9 were calculated as pg/mL.

### 4.9. Western Blotting for Proteins

The western blotting was performed in striatal homogenates of each experimental group. Striatal sections were used because the loss of dopaminergic nigral end terminals mostly affected the striatum. Western blotting was carried out for the samples isolated from striatal tissues to measure the expression of specific proteins. The samples were homogenized with phosphatase and protease inhibitors in RIPA buffer in a 1:10 ratio weight by volume. Then, 35 µg of protein from each sample after quantification of proteins was loaded in SDS-PAGE and the gel was run using the trans-turbo BIO-RAD technique. The proteins from gels were electrotransferred to the PVDF membrane. The PVDF membranes were blocked with 5% nonfat dried milk in PBST and incubated on a vertical shaker for 1 h. The membranes were washed with PBST after blocking and incubated at 4 °C overnight with primary antibodies at dilutions of α-synuclein (1:1000), COX-2 (1:1000), iNOS (1:1000), Bax (1:2000), Bcl-2 (1:500), mTOR (1:1000), phosphor-mTOR (1:1000), LC3B (1:1000), and p70s6K (1:1000). The membranes were washed with PBST, and the corresponding secondary antibodies were added and incubated for one hour. The membranes for protein bands were subjected to the Chemiluminescence West Pico detection Kit (Thermo Fisher Scientific, Waltham, MA, USA). Using Image-J (NIH, Bethesda, MD, USA), the observed bands were quantified.

### 4.10. Estimation of Microglia and Astrocyte Activation by Immunofluorescence Staining

The brain was collected from each experimental group following intracardiac perfusion and frozen and fixed in paraformaldehyde for 48 h. The brains were treated with 30% sucrose for three consecutive days. The brains were vertically sectioned and the bregma position was taken as zero. Astrocytes with GFAP and microglia with Iba-1 were stained using striatal sections from all experimental groups. The 30-micrometer-thick sections were precisely sectioned using the procedure of the floating sections. The sections were blocked with a blocking reagent composed of 10% normal goat serum in PBS, 0.3% Triton-X100, and 1% BSA for one hour. After washing, the sections were dispensed with GFAP (1:1000) and Iba-1 (1:1000) and incubated overnight at 4 °C. The sections were washed with PBS, and the secondary fluorescent secondary antibody (Alexa 488), specific to the respective primary, was dispensed and incubated for one hour. After washing twice with PBS, the sections were mounted with a fluorescent vector stain incorporated with DAPI. The sections were viewed in a fluorescent microscope at 20× (Nikon Eclipse Ni). The activated astrocytes and microglia were counted in each brain, from a minimum of three coronal slices of the respective same striatum levels. The images were quantified using the Image-J software (V 1.61) after being randomly divided into three equal-sized fields (NIH, Bethesda, MD, USA). The area of interest was circled, and measurements of the area, circularity, and mean fluorescence were performed, while considering a variety of nearby background readings. The formula: TCCF = integrated density − (area of selected cell × mean fluorescence of background readings), was used to calculate the total corrected cellular fluorescence (TCCF). The individual numbers were used for statistics. To avoid calculation bias, each reading was determined by an observer who was not familiar with the experimental procedures. A total of seven images per group were used for data analysis. The value is shown as a percentage of the control.

### 4.11. Assessment of Tyrosine Hydroxylase (TH-Staining) for Dopaminergic Neurons in Substantia Nigra and Striatum

The brain was collected from each experimental group following intracardiac perfusion and frozen and fixed in paraformaldehyde for 48 h. The brains were treated with 30% sucrose for three consecutive days. The brains were vertically sectioned and the bregma position was taken as zero. Cryoprotected rat brains were serially sectioned (30 nm) using a cryostat (Leica, Wetzlar, Germany). The serial sections of the substantia nigra were washed twice with PBS and inactivated with 1% H_2_O_2_. The sections were washed twice with PBS and blocked for an hour with blocking buffer (10% normal goat serum in PBS with 0.3% Triton-X 100). The sections were stained with goat anti-rabbit tyrosine polyclonal antibody after blocking and incubated overnight. The striatal sections were washed twice with 1% PBS and incubated for one hour with a 1:1000 dilution of a biotinylated secondary antibody. To assess the TH immunoreactivity, the sections were stained and developed with an avidin-biotin-peroxidase complex system (ABC kit, CA 94560, Mowry Avenue, Newark, NJ, USA), preceded by 3,3′ diaminobenzidine (DAB). The sections were mounted on electrophilic slides using DPX media. The representative images were then captured by the camera attached to the light microscope and the images were evaluated. A total of seven images per group were used for data analysis. The value is shown as a percentage of the control.

### 4.12. Assessment of Loss of TH-ir Dopaminergic Neurons and TH-ir Dopamine Nerve Fibers

In the SNc region, the loss of nerve fibers was investigated by assessing the loss of TH-immunoreactive (TH-ir) neurons in the stained sections. In short, the sections from level three to level five of the medial terminal nucleus regions were counted. As a percentage of naive control, the counts were displayed [13,14,15]. The optical density of TH-ir dopaminergic fibers in the striatum to investigate striatal fiber loss was calculated using Image-J software V1.61. From each group, the optical densities of TH-ir from three different sections were collected, and the measured densities were expressed as a percentage of the naive control. A total of seven images per group were used for data analysis. The value is shown as a percentage of the control.

### 4.13. Protein Estimation

The Pierce BCA protein assay kit was acquired from Thermo Fisher Scientific (Waltham, MA, USA) and was used for finding the protein concentration in all samples of respective groups, following the manufacturer’s instructions.

### 4.14. Statistical Analysis

The data are presented as mean ± SEM (standard error of the mean) and analyzed by one-way analysis of variance and the post hoc Tukey’s test. Using the SPSS12 program, the criterion of statistical significance between different experimental groups was determined. The statistical significance was set at *p* < 0.05.

## Data Availability

The data used to support the findings of this study are already incorporated in the Results Section.
